# Medication Adherence in the General Population

**DOI:** 10.1371/journal.pone.0050537

**Published:** 2012-12-18

**Authors:** Julia A. Glombiewski, Yvonne Nestoriuc, Winfried Rief, Heide Glaesmer, Elmar Braehler

**Affiliations:** 1 University of Marburg, Department of Clinical Psychology and Psychotherapy, Marburg, Germany; 2 University of Leipzig, Department of Medical Psychology and Medical Sociology, Leipzig, Germany; 3 University of Hamburg, Department of Clinical Psychology and Psychotherapy, Hamburg, Germany; The University of Hong Kong, Hong Kong

## Abstract

**Background:**

Adherence to medication is low in specific populations who need chronic medication. However, adherence to medication is also of interest in a more general fashion, independent of specific populations or side effects of particular drugs. If clinicians and researchers expect patients to show close to full adherence, it is relevant to know how likely the achievement of this goal is. Population based rates can provide an estimate of efforts needed to achieve near complete adherence in patient populations. The objective of the study was to collect normative data for medication nonadherence in the general population.

**Methods and Findings:**

We assessed 2,512 persons (a representative sample of German population). Adherence was measured by Rief Adherence Index. We also assessed current medication intake and side effects. We found that at least 33% of Germans repeatedly fail to follow their doctor's recommendations regarding pharmacological treatments and only 25% of Germans describe themselves as fully adherent. Nonadherence to medication occurs more often in younger patients with higher socioeconomic status taking short-term medications than in older patients with chronic conditions. Experience with medication side effects was the most prominent predictor of nonadherence.

**Conclusions:**

The major strengths of our study are a representative sample and a novel approach to assess adherence. Nonadherece seems to be commonplace in the general population. Therefore adherence cannot be expected per se but needs special efforts on behalf of prescribers and public health initiatives. Nonadherence to medication should not only be considered as a drug-specific behaviour problem, but as a behaviour pattern that is independent of the prescribed medication.

## Introduction

The World Health Organisation identified medication non-adherence as one of the major causes of morbidity, mortality and health care costs [1]. It is estimated that between 30 and 50% of prescribed medication, depending on the disease and the health care system, is not taken as directed [2], [3].

Numerous researchers put effort into examining the rates and predictors of adherence to contribute to the development of adherence boosting interventions [4]–[7]. These studies usually focus on specific medications in particular populations, such as HAART adherence in HIV-positive individuals [8] or blood glucose lowering medications in diabetes patients [9]. The results of these studies cannot be generalized to other populations as well as other drugs.

However, adherence to medication is also of interest in a more general fashion, independent of specific populations or side effects of particular drugs. If clinicians and researchers expect patients to show close to full adherence, it is relevant to know how likely the achievement of this goal is. Population based rates can provide an estimate of efforts needed to achieve near complete adherence in patient populations.

Therefore the issue of adherence in general raises a couple of important questions, which can only be answered with appropriate investigations in the general population:

How many people, regardless of suffering from a chronic condition or just having a common cold from time to time, generally follow their doctor's advice?
What are the base rates for adherence that can be expected in the general population?
How certain can a family doctor be that an average patient will show adherent behaviours after leaving their office?
What are the factors generally associated with adherence?
Is nonadherence a drug specific behaviour or rather a behavioural pattern?
And, last but not least: what is “normal adherence” with regards to the general population?

Only a fistful of studies have assessed adherence to medication independently from a specific drug or a particular population. Bardel et al., for example, assessed adherence to “prescribed drugs” in women in central Sweden [10] concluding that “the same women had different degrees of adherence to different medication”. Yeaw et al. [1] investigated adherence to medication for six different chronic medication classes, reporting “variable but uniformly suboptimal medication use”. Briesacher et al. [11] compared drug adherence rates among patients with different medical conditions, concluding that adherence varied by disease. Studies like these are important to learn more about general behaviours and attitudes concerning medication. However, useful information on general adherence in the population can only be obtained with sufficiently representative study samples. Although providing large sample sizes, none of the previous studies have actually included representative samples, thus limiting the informative value of the reported results.

The aim of the present study is to describe and categorize general adherence to medication in a representative German sample. The study is of novelty in its field because it focuses on neglected questions in adherence research:

First, to our knowledge, this is the first study representatively assessing adherence to medication in the general population
Secondly, adherence was assessed independently of medication groups as a behavioural disposition
Thirdly, we will identify predictors of nonadherence to medication in a representative sample

The results from a population based study on adherence may open new field of research that focuses on general behavioural patterns associated with non adherence. Based on this research behavioural trainings for patients and their doctors could be developed in order to improve adherence to medication independently from specific drugs.

## Methods

### Procedure

A representative sample of the German general population was selected with the assistance of a demographic consulting company (USUMA, Berlin, Germany). The population based survey met the ethical guidelines of the international Code of Marketing and Social Research Practice by the International Chamber of Commerce and the European Society for Opinion and Marketing Research. The market research company conducting data sampling is member of a group with general ethical approval from the government to conduct these types of surveys. According to the Federal Data Protection Act (Bundesdateschutzgesetz BDSG, § 30a), the need for consent from a specific ethics comitee is waived for USUMA surveys. A total of 2,512 people agreed to participate and signed written informed consent forms.

The area of Germany was separated into 258 sample areas representing the whole country. After selecting a sample area, households of the respective area and members of these households fulfilling the inclusion criteria were selected using the Kish-selection-grid technique. The Kish-selection-grid technique is aimed at sampling individuals on the doorstep among household residents. The system is devised so that all individuals in a household have an equal chance of selection. The sample was aimed to be representative in terms of age, gender, and education based on data from German Federal Bureau of Statistics on German population from 2009 [12]. Only people older than 13 years were included. A first attempt was made for 4,572 valid addresses; 378 selected persons were not present even after 3 attempts of visiting them, 864 households rejected participation in general, while 682 target persons did not agree to participate. Because an additional 12 persons did not answer major parts of the interview, the final sample consisted of 2,512 persons.

### Measures

#### Adherence measure

Adherence to medication was assessed using a four-item self-report scale, the Rief Adherence Index (RAI). The participants were questioned on their general past and present behaviours concerning medication intake, independently of current medication intake. The participants were instructed to consider “all past behaviours concerning any prescribed medication” in order to assess a behaviour pattern that is independent of the prescribed medication.

The RAI consists of 4 items:

1. I stored or threw away prescribed medication without unwrapping it
2. I changed the doses of my medication without doctor's authorisation depending on my well-being
3. I discontinued my medication earlier then the doctor recommended
4. I discontinued my medication because of mild side-effects

Respondents were instructed to indicate their agreement with each statement on a five-point Likert scale. Item responses were:

1 = (almost) never happened (in 0–20% of cases)

2 = rarely happened (in 20–40% of cases)

3 = often happened (in 40–60% of cases)

4 = happened most of the time (in 60–80%)

5 = (almost) always happened (in 80–100% cases).

The RAI's maximum score is 20, reflecting reports of generally very high non-adherence, the minimum score is 4, reflecting reports of high adherence to prescribed medication with non-adherent behaviours in under 20% of cases.

The use of percentage scores is a novelty and allows an estimation of the health-economic relevance of nonadherece.

#### Current drug intake

Participants were asked whether they were currently taking any medications and if so, the type of medication taken. Items included the ten most common drug classes (anti-diabetic drugs, pain killers, lipid-lowering drugs, antidepressants, antihypertensives, asthma medication, contraceptives, antibiotics, tranquilizers, and sleep medication [13].

#### General Assessment of Side Effects GASE

The GASE [14] is a structured, systematic screening tool assessing the general burden of side effects from concurrent medication. In addition to reporting symptoms of all body parts and their relation to current medication intake, participants were requested to rate the general severity of current side effects on a four-point Likert scale from 0 (“not present”) to 3 (“severe”). This rating was used for our analyses.

#### Demographic variables

Age, gender and monthly income were demographic variables of interest for our analyses.

### Statistics

Factor analysis and internal consistency analysis were performed to validate the factor structure and consistency of RAI. Descriptive statistics were carried out to describe the distribution of adherence to medication in the general population. A linear regression analysis was chosen to assess the predictors of adherence to medication. All analyses were carried out with SPSS 17 for Windows ™.

### Ethical approval

The population based survey met the ethical guidelines of the international Code of Marketing and Social Research Practice by the International Chamber of Commerce and the European Society for Opinion and Marketing Research.

## Results

### Demographic characteristics of the sample

The mean age of the final sample was 49.4 years (SD 18.2), and 55.8% of the total sample was female. 53% had more than 9 years eduacation. Table 1 compares the data of the study with the German population according to “Statistisches Bundesamt 2009” (German Federal Bureau of Statistics) [12]. The age group 61–70 was slightly overrepresented, and the oldest age group (>70) was underrepresented in our study. The percentages of subjects with 12 and more years of education were as follows in this sample: 23% (18–30 years); 20% (31–40 years); 22% (41–50 years); 17% (51–60 years); 10% (61–70 years), and 9% (>70 years). Compared to the micro census of Germany, these data confirm representativeness of our sample. The final sample consisted of 2,512 persons. 2,452 reported past or current prescribed medication intake. 6.8% reported current intake of anti-diabetic drugs, 22.4% pain killers, 9% lipid-lowering drugs, 1.9% antidepressants, 20.9% antihypertensives, 2.7% asthma medication, 6.8% contraceptives, 2.9% antibiotics, 6.3% tranquilizers/sleep medication and 8.8% other drugs. 34.4% of the sample reported that drug intake was necessary for their health. On average participants were prescribed .9 (SD 1.1) drugs. 0.7% of patients reported prescription of 5 or more different drugs. The majority of the participants reported prescription of one (27%) or two (15%) different drugs.

**Table 1:** Comparison of the study data with the German population according to “Statistisches Bundesamt 2009”.

	Males (in %)		Females (in %)	
Age	Study	German pop.	study	German pop.
18–30	17.2	18.1	15.7	16.2
31–40	16.0	15.8	18.3	14.4
41–50	21.2	21.5	20.0	19.4
51–60	17.8	17.0	16.9	16.1
61–70	17.3	13.7	16.6	13.7
>70 y.	10.4	13.9	12.5	19.8

### Rief Adherence Index (RAI)

Cronbach's Alpha for RAI was *r* = .79, indicating satisfactory internal consistency. An exploratory factor analysis validated a one-factor solution with communalities from .56 to .72 and 62% of variance explained by the single factor “adherence”. This confirms that one common factor explains major parts of non-adherent behaviours.

RAI was validated by Pearson's r correlations with a German version of the Beliefs about Medicines Questionnaire (BMQ) [15] and the German Version of Somatosensory Amplification Scale (SSAS) [16] (*n* = 2512). As expected, RAI (with higher scores indication higher non-adherence) showed the highest correlation with the BMQ General Harm Scale (*r* = .24, *p* = .000). High scores on the General Harm Scale represent negative views about medicines.

There was also positive correlations of RAI with the BMQ General Overuse Scale (*r* = .17, *p* = .000). High scores on the General Overuse Scale indicate negative views about the way in which medicines are prescribed. As expected, there was also a significant correlation with the SSAS (*r* = .12 *p* = .000) indicating higher attention for interoceptive sensations among participants with higher non-adherence. Accordingly, RAI correlated negatively with BMQ Utility of Medicines Scale (*r* = −.08 , *p* = .000). This correlation implies that participants reporting more non-adherence stated that medicines were less useful to improve their health. Altogether, those results suggest that RAI is a reliable and valid measure of adherence.

### Subjective severity of side effects

The average severity of current side effects measured by GASE on a four-point Likert scale from 0 (“not present”) to 3 (“severe”) was .43 (SD = .68).

### Distribution of adherence to medication in the general population

**Figure 1:**
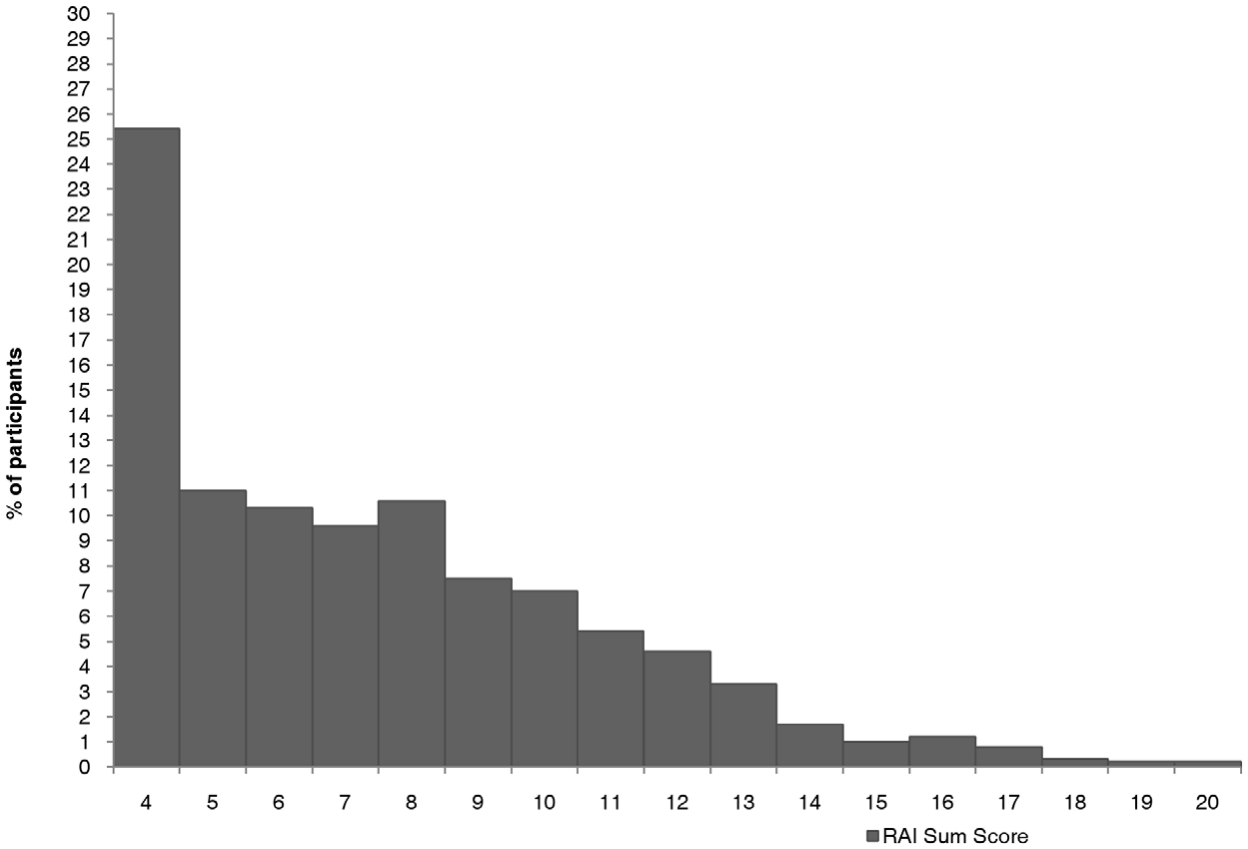
Distribution of RAI sum scores (N = 2,452).

**Figure 2:**
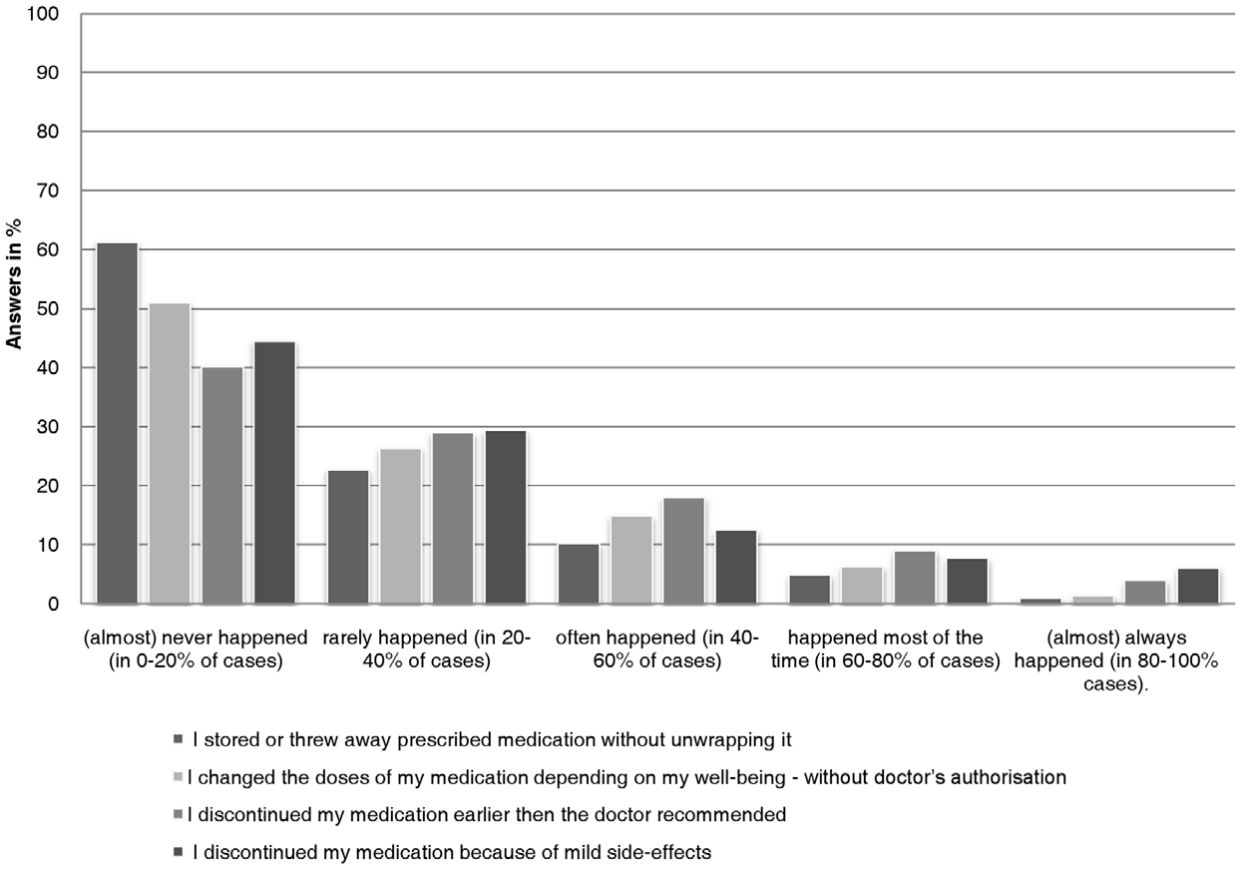
Distribution of responses to single RAI items (N = 2,452).

2,452 of 2,512 participants responded to all 4 items of RAI with a mean of 7,5 (SD = 3,35). The distribution of the sum scores of RAI is presented in Figure 1, the distribution of responses to RAI items in Figure 2. Based on these statistics, a RAI sum score of 8 was defined as a cut-off between adherent and non-adherent medication intake. This cut-off defines participants that report adherent behaviours in at best 60–80% of cases as generally rather adherent to medication, corresponding with the verbal anchor of “rarely happened” as response to all non-adherent behaviours and a RAI sum score of maximally 8. Accordingly, participants reporting non-adhere behaviours in more than 40% of cases (and having a RAI sum score of 9 and more) would be considered as less adherent to medication.

According to the distribution of RAI sum scores as displayed in Figure 1, 25.4% (n = 622 participants) of the responders reported very high adherence to medication with non-adherent behaviours in maximally 20% of cases. Only a minority of participants (0.2%, n = 6 participants) reported all non-adherent behaviours in 80–100% of cases for all items, reaching the maximum sum score of 20.

With respect to the defined cut-off, 66.8% (1638 of 2452 participants) reported to be generally rather adherent to medication, while 33.2% (814 of 2452 participants) reported to be generally rather non-adherent, acting contrary to their doctor's recommendation in 40–100% of cases.

### Predictors of non-adherence in the general population

1,283 of 2,452 participants (51.1%) reported to be “currently taking medication”. Age, gender and socioeconomic status (represented by monthly household income), current intake of different medication classes (anti-diabetic drugs, pain killers, lipid-lowering drugs, antidepressants, antihypertensives, asthma medication, contraceptives, antibiotics, tranquilizers, and sleep medication) and general intensity of side-effects were considered as potential predictors of adherence to medication.

A linear regression analysis (*n* = 1.256 patients who reported to be taking medication and provided complete data) showed that higher adherence was associated with reports of less intensive side effects (*ß* = .18, *t* = 6.39, *p*<0.001, *partial r* = .18), higher age (*ß* = −.18, *t* = −5.66, *p*<0.001, *partial r* = −.16), less monthly income (*ß* = .09, *t* = 3.1, *p*<0.05, *partial r* = .09), female gender (*ß* = −.06, *t* = −2.36, *p*<0.05, *partial r* = −.07), higher current usage of antihypertensives (*ß* = −.11, *t* = −3.52, *p*<0.001, *partial r* = −.1) and less current usage of pain killers (*ß* = .08, *t* = 2.9, *p*<0.05, *partial r* = .08) and antibiotics (*ß* = .06, *t* = 2.35, *p*<0.005, *partial r* = .07) with a total *R^2^* = .12 (*F* (7, 1255) = 23.6, *p*<0.001)).

All other variables were excluded through the forward procedure during the regression analysis.

## Discussion

### Discussion of principal findings

This study is the first to address the neglected subject of adherence to medication in the general population. We found that at least 33% of Germans repeatedly fail to follow their doctor's recommendations and only 25% of Germans describe themselves as fully adherent.

At first sight this result appears familiar – nonadherence rates of 30–40%, depending on the definition and assessment of adherence as well as type of drug and disease, are commonly described [1], [3]. However, these rates are reported only for potentially problematic populations, such as HIV-positive drug users [17] and for chronic medication classes such as oral antidiabetics [18] or statins [19].

Our results show that these nonadherence rates are not drug specific but resemble a more general behavioural pattern.

The data presented here clearly show that full medication adherence is uncommon. Only 25% of the German population report following or having followed their doctor's advice in over 80% of cases and thus meet the definition of “full adherence” [17]. A substantial proportion of the representative sample reported to repeatedly store or throw away prescribed medications, to change the dosages without doctor's authorisation or to discontinue medication earlier than recommended. Because we used subjective, retrospective measures of adherence that are prone to biases such as social desirability or memory effects and chose a liberal cut-off, the actual nonadherence rates might be even higher than presented here. This is rather surprising considering that one of the main barriers to adherence, out-of-pocket costs for medication [5], [20] is unlikely an influential factor of nonadherence in Germany. Most of the patients in Germany have health plans with no or only marginal cost sharing for medication.

Thus, our results indicate that high nonadherence to medication is not only a problem of specific population, medication class or costs: an average German person has the same probability of nonadherent behaviours concerning prescribed medication as, for example, an HIV positive drug addict [21]. Trying to achieve higher medication adherence in specific populations might be a challenging task since nonadherence is common in general population.

In addition to rates of medication adherence in the general population, we also investigated predictors of adherence in a subgroup of participants who reported to take medication at the time point of the survey.

We found that experiences with side effects limited medication adherence. Our results indicate that younger males with higher socioeconomical status fail to follow their doctor's recommendations more often than older, less well situated females. Additionally, we showed that patients with long-term medication regiments like antihypertensive treatment were more likely to take their medication as prescribed. Patients with short-term prescriptions such as antibiotics were more likely to ignore doctor's advice or patient information sheets and thereby contributing to the international public health challenge of antimicrobial resistance [22].

### Comparison with other studies

These results are in line with the conclusions from another population-wide study: Bardel et al. [10] investigated around 3.000 Swedish women and found that adherence to medication was the lowest amongst young women who regarded their medication as unimportant. Accordingly, Briesacher et al. [11] found that an age younger than 60 years was associated with lower adherence across different diseases and that individuals with hypertension achieved highest adherence rates. The WHO [13] reported that one of the main barriers to adherence were side effects. Higher individual expectation and experience of side effects have been shown to predict nonadherence (e.g., patient-initiated changes in prescribed medication) across a wide range of diseases [23]–[25]. Our study is in line with this conclusion, with experienced severity of side effects showing the highest correlation with nonadherence. Therefore, negative expectations concerning side effects should be assessed whenever medication is prescribed.

### Limitations and strengths of the study

Our study has several limitations. A potentially controversial point is the assessment of adherence. Self reports can be susceptible to errors, generally overestimating patient's adherence [26]. On the other hand there are no other valid methods than questionnaires to assess adherence as a behavioural disposition in a large representative sample, independently of current medication intake. Accordingly, authors who discussed several methods of measuring adherence came to the conclusion that “Even today, patient's self reports can simply and effectively measure adherence” [26].

A potential other limitation is that we did not differentiate between adherence and persistence as requested by some authors [27]. We decided to do so guided by results of a statistical analysis: although we included questions about adherence and persistence behaviours, the factor analysis of RAI clearly suggested one factor. However, consecutive validation studies of RAI are needed to confirm this result.

A further limitation of our study is that we only assessed a selection of possible predictors of adherence. The explained variance in adherence was 12%, similar to what was found in numerous other predictor analyses [4]. In general, authors agree that the ability to explain adherence remains poor [28], although some robust predictors were confirmed in our study. Nevertheless, several potentially important predictors were not included. We believe that we could contribute to the literature by describing the impact of the experienced severity of side effects, age, gender, chronic diseases and socioeconomic status on adherence. Nonetheless, the results can only be interpreted while keeping in mind that further patient factors such as clinical depression, contextual factors such as complexity of treatment, health care characteristics and, last but not least, patient-clinician relationship factors also contribute to the explanation of the complex issue of medication nonadherence. Our data show that nonadherence is not only dependent on these contextual factors but is rather a behavioural pattern.

The adherence cut-off chosen for this study might be regarded as rather liberal. Some studies define optimal adherence as 100% uptake of prescribed doses and the most liberal cut-offs used in various studies define uptake greater than 80% as “adherence” [17]. For RAI this means that sum scores higher than 4 would be regarded as adherent in less than 80% of cases. Correspondingly, only 25% of our sample show full medication adherence given this more conservative criterion. On the other hand, adverse event risks associated with over or under dosage of specific drugs depend on effective levels of those drugs. Hence adherence cut-offs might best be defined separately for different medication classes. Since we tried to assess “normal” adherence as a behavioural disposition in a population taking different drugs or no drugs at all, we decided to choose a more liberal cut-off based on a mean of the general population's behaviour.

Although the sample is representative in comparison to the data from German Federal Bureau of Statistics, one possible limitation is a risk of bias since a part of approached households did not agree to participate.

The major strengths of our study are a representative sample and a novel approach to assess adherence. RAI's items additionally allow quantifying nonadherence percentagewise and thus allowing for an estimation of the economic costs associated with discontinuation of prescribed medications.

### Conclusion

These results show that nonadherence is neither a problem of specific patients nor of drug classes. Despite several papers on predictors of adherence there is no study that could explain a sufficient amount of variance [4]. Thus, nonadherence can be considered as partly independent of contextual factors that are commonly addressed in adherence enhancement programmes. The problem of nonadherence could be better solved by considering stable behaviour patterns concerning medication intake as well as individual nonadherence risk factors such as personal history of experiences with side effects. Further research on adherence and adherence enhancement programmes should focus on those behaviour patterns and on patients' expectations concerning side effects.
